# Mycotoxin Residues in Chicken Breast Muscle and Liver

**DOI:** 10.3390/foods14122017

**Published:** 2025-06-07

**Authors:** Tina Lešić, Jelka Pleadin, Nina Kudumija, Dora Tomašković, Ana Vulić

**Affiliations:** 1 Laboratory for Analytical Chemistry, Croatian Veterinary Institute, Savska Cesta 143, 10000 Zagreb, Croatia; pleadin@veinst.hr (J.P.); kudumija@veinst.hr (N.K.); vulic@veinst.hr (A.V.); 2 Laboratory for Food Microbiology, Croatian Veterinary Institute, Savska Cesta 143, 10000 Zagreb, Croatia; stojevic@veinst.hr

**Keywords:** aflatoxins, ochratoxin A, cyclopiazonic acid, sterigmatocystin, citrinin, poultry, meat, LC-MS/MS, contaminants

## Abstract

The global increase in chicken meat production and consumption has heightened concerns regarding the safety of chicken meat and its derived products. This study aimed to investigate the presence of *Penicillium* and *Aspergillus* mycotoxins in 50 samples of chicken breast muscle and liver collected from the Croatian market. Eight mycotoxins commonly produced by *Aspergillus* and *Penicillium* species were analyzed: aflatoxins B_1_ (AFB_1_), G_1_ (AFG_1_), B_2_ (AFB_2_), and G_2_ (AFG_2_); sterigmatocystin (STC); ochratoxin A (OTA); cyclopiazonic acid (CPA); and citrinin (CIT). Mycotoxin concentrations were determined using liquid chromatography–tandem mass spectrometry (LC-MS/MS) following sample cleanup with immunoaffinity columns while a QuEChERS-based method was applied for CPA. Mycotoxin occurrence was higher in liver samples, indicating the liver as primary site of mycotoxin accumulation compared to muscle tissue, where only CPA was detected. CPA was present in 20% of all samples, with the highest concentration (6.50 µg/kg) found in breast muscle, detected for the first time in fresh meat. AFB_1_ and OTA were each detected in 10% of samples, and CIT was found in 4%—all exclusively in liver tissue. Notably, 4 out of the 17 contaminated samples contained more than one mycotoxin. Although the detected concentrations can be considered too low to pose an immediate health risk, the contamination rate suggests further research into these mycotoxins in chicken and other poultry species is needed.

## 1. Introduction

Mycotoxins, a heterogeneous group of toxic secondary metabolites, are primarily produced by fungi from the genera *Aspergillus*, *Penicillium*, and *Fusarium*. Some of the most important mycotoxins produced by *Aspergillus* and *Penicillium* species are aflatoxins (AFs), OTA, CIT, STC, and CPA [1]. Mycotoxin contamination of food and feed poses risks to both human and animal health due to their carcinogenic and toxic properties [2]. Aflatoxins (AFs), mainly produced by *Aspergillus flavus* and *Aspergillus parasiticus*, are among the most toxic mycotoxins, with AFB_1_ classified by the International Agency for Research on Cancer (IARC) as a Group 1 human carcinogen [3]. STC, an emerging mycotoxin produced by *Aspergillus* sp., is a biosynthetic precursor of AFB_1_. Although less potent, it shares similar toxic effects and is considered a potential human carcinogen [4,5]. Due to a lack of data, the European Food Safety Authority (EFSA) Panel on Contaminants in the Food Chain (CONTAM) recommends monitoring STC in food and feed using sensitive methods [6]. Some *A. flavus* strains can produce both AFs and CPA, raising concerns about co-occurrence and combined exposure, which the EFSA CONTAM Panel highlighted as important for assessment [7]. Although relatively unexplored as an emerging mycotoxin, CPA has been found in significant levels in pig meat products [4,8,9,10]. OTA, classified as a possible human carcinogen (Group 2B) by the IARC, is one of the most prevalent and toxic mycotoxins found in meat products [10,11]. CIT can co-occur with OTA and may exert additive or synergistic toxic effects. It is produced by *Aspergillus*, *Monascus*, and *Penicillium* species, notably *Penicillium citrinum*, one of the most widespread food-contaminating fungi globally [10].

Global chicken meat production and consumption are steadily rising, driven by improved availability, changing diets, market expansion, and its high nutritional value at a low cost. Among all livestock sources, poultry—particularly chicken—has emerged as a cornerstone of sustainable food systems, offering efficient feed conversion, a relatively low environmental impact, and strong nutritional benefits, making it a highly favorable protein source in the context of global food security [12]. In addition, chicken liver is a rich, natural source of heme iron; so, the inclusion of chicken liver in children’s diets has been increasingly recognized as a low-cost strategy for preventing iron deficiency anemia in children [13]. Additionally, in Croatia, both poultry production and consumption have been rising. In 2019, poultry meat production reached approximately 53,900 metric tons, a 3% increase from the previous year. Per capita consumption rose to 19 kg in 2021, a 14% increase compared to the previous year, although still below the EU average of 22 kg [14,15].

Mycotoxin contamination in chicken meat and derived products can occur via two primary pathways. The first involves the ingestion of contaminated feed by animals, leading to the accumulation of toxin residues in edible tissues. Mycotoxins are metabolized in the liver, kidneys, and digestive tract, and can be readily transferred to poultry products, including meat and eggs, depending on the type of mycotoxin and poultry species, among other factors [16,17]. The second pathway relates to contamination during processing, preservation, and distribution [18]. Mycotoxins commonly enter animal feed via fungal contamination, and feed remains the major source of animal exposure—ultimately leading to human exposure [19]. With the ongoing effects of climate change, the risk of mycotoxin contamination in animal feed is expected to increase. Evidence suggests that climate change may negatively impact global crop production, with rising temperatures favoring thermotolerant fungal such as *Aspergillus* sp. [20]. Currently, there is no specific legislation regulating the maximum levels (MLs) of mycotoxins in meat and meat products [21].

Previous studies have reported mycotoxins in poultry feed [19,22] as well as in chicken meat and liver, with a primary focus on AFs, OTA, zearalenone (ZEN), and deoxynivalenol (DON)—the latter two produced by *Fusarium* sp. [23,24,25,26,27,28]. Given the high demand for poultry meat and its nutritional importance (particularly for children), ensuring the safety of these products, including monitoring for naturally occurring mycotoxin contamination, is of a great importance. To date, no comprehensive studies have examined mycotoxin residues in poultry meat and organs in Croatia, and data on some mycotoxins, such as CPA, are generally lacking. However, identifying new sources of mycotoxin exposure is critical for accurate health risk assessments. This study aims to collect initial data on the presence and contamination rates of *Penicillium* and *Aspergillus* mycotoxins AFs, OTA, CIT, STC, and CPA in chicken breast muscle and liver, as a starting point for more comprehensive investigations in chicken and other poultry species.

## 2. Materials and Methods

### 2.1. Samples

A total of 50 samples—25 of chicken breast muscle and 25 of chicken liver—were collected from local small-scale Croatian producers at markets and family farms between March and September 2024. The samples were sourced from 16 different producers across three Croatian regions: eastern, central, and northern. The samples, both breast muscle and liver, were obtained fresh in commercially available packaging, each with a weight of approximately 500 g. All liver and breast muscle samples originated from different individual chickens and different producers, ensuring source diversity. Detailed information for each analyzed sample is provided in Table S1.

All samples were homogenized using a Grindomix GM 200 laboratory homogenizer (Retsch, Haan, Germany) and stored in plastic containers at −18 °C until mycotoxin analysis.

### 2.2. Standards and Reagents

Mycotoxin standards, including a mixture of aflatoxins (AFB_1_, AFB_2_, AFG_1_, and AFG_2_) at 1 µg/mL in acetonitrile (Art. No. DRE-A30000021AL), OTA at 10 µg/mL in acetonitrile (Art. No. DRE-A15670000AL-10), CIT at 100 µg/mL in acetonitrile (Art. No. DRE-A11668522AL-100), CPA at 100 µg/mL in acetonitrile (Art. No. DRE-A11833700AL-100), and STC at 50 µg/mL in acetonitrile (Art. No. DRE-V16974700AL-50), were purchased from LGC Standards (Wesel, Germany). Working solutions were freshly prepared on the day of analysis. Ultrapure water was obtained using a Direct-Q^®^ 3 UV water purification system (Merck, Burlington, MA, USA). Acetic acid, methanol, acetonitrile, and ammonium acetate (all HPLC grade) were obtained from Honeywell (Charlotte, NC, USA). All other reagents, including Tween 20, were of analytical grade and purchased from Sigma-Aldrich (St. Louis, MO, USA).

### 2.3. Mycotoxins Extraction Procedure

The extraction of aflatoxins (AFB_1_, AFG_1_, AFB_2_, and AFG_2_), OTA, STC, and CIT was performed following the method previously described by Lešić et al. [8], based on the instructions provided by the immunoaffinity column manufacturer (R-Biopharm Rhône Ltd., Glasgow, UK). Briefly, samples were extracted with 20 mL of 80% methanol, vortexed, and centrifuged. The supernatant was filtered, diluted with 0.1% Tween 20 in phosphate-buffered saline (PBS, pH 7.4) at a 1:5 ratio, and passed through immunoaffinity columns. Aflatoxins and OTA were purified using AFLAOCHRAPREP^®^ columns, CIT using Easi-extract CITRININ^®^, and STC using Easi-extract STERIGMATOCYSTIN^®^ columns. After sample loading, columns were rinsed with PBS (pH 7.4), and mycotoxins were eluted using methanol followed by ultrapure water.

The extraction of CPA from chicken samples was performed according to the method described by Vulić et al. [29], using rOQ QuEChERS extraction packets (Phenomenex, Torrance, CA, USA). Briefly, 5 mL of 25% acetic acid was added to each sample, followed by 5 mL of acetonitrile. The mixture was shaken on a rotary shaker for 30 min. After the addition of rOQ QuEChERS extraction salts, the samples were centrifuged and the supernatant was filtered through 0.2 µm PTFE syringe filters.

### 2.4. LC-MS/MS Analysis

Mycotoxins were analyzed using liquid chromatography–tandem mass spectrometry (LC-MS/MS) on a high-performance liquid chromatograph (HPLC) (1260 Infinity, Agilent Technologies, Santa Clara, CA, USA) coupled with a triple quadrupole mass spectrometer equipped with an electrospray ionization (ESI) source (6410 QQQ, Agilent Technologies, Santa Clara, CA, USA). Chromatographic separation was performed on a C18 reverse-phase analytical column (Gemini, 150 × 4.6 mm, 5 µm particle size; Phenomenex, Torrance, CA, USA) coupled with a SecurityGuard™ Gemini C18 pre-column cartridge (4 × 3.0 mm ID; Phenomenex, Torrance, CA, USA). Chromatographic and instrumental conditions were previously described by Lešić et al. [8] and Vulić et al. [29], respectively. For quantification, one precursor ion and two product ions were monitored for each mycotoxin (Table S2).

### 2.5. Method Validation

Method validation was conducted according to the procedures described earlier by Lešić et al. [8], with the exception of aflatoxins B_2_, G_1_, and G_2_, which are reported here for the first time. The method performance characteristics are summarized in Table 1. Solvent calibration was used within specific concentration ranges for each mycotoxin, as shown in Table 1, to assess linearity. The matrix effect was evaluated by comparing the peak areas of each mycotoxin in standard solutions with those in blank matrix samples spiked after sample preparation at the same concentration levels. Limits of detection (LOD) and quantification (LOQ) were calculated based on the slopes of the calibration curves and the signal intensities obtained from 10 meat product samples spiked at target LOD levels. Recovery was determined by analyzing 10 meat product samples spiked at known levels.

### 2.6. Statistical Analysis

Statistical analyses were performed using SPSS Statistics Software version 22.0 (IBM Corp., Armonk, NY, USA). The Shapiro–Wilk test was applied to assess the normality of data distribution. To determine the statistical significance of differences in CPA concentrations between liver and muscle samples, the Independent Samples *t*-test was employed. Statistical significance was set at *p* < 0.05. 

## 3. Results and Discussion

The quality of poultry feed and feeding practices is a major contributor to the contamination of poultry food products [19]. Mycotoxin occurrence in feed can be influenced by climatic conditions, which vary annually across different geographical regions, leading to fluctuations in contamination levels across countries and years. In Croatia, OTA and CIT were sporadically detected in maize, with CIT found in up to 38% of samples (max. 968.6 µg/kg) and OTA in 6% (max. 6.0 µg/kg) [30]. AFB_1_ was most frequently detected in 2012 and 2021, with up to 70% of maize samples contaminated and concentrations reaching as high as 422.2 µg/kg, particularly during drought-affected seasons [30]. Studies have proven that feeds commonly have species from *Aspergillu*s and *Penicillium* genera as dominant flora [31]. Among the mycotoxins investigated in this study, the EU regulates AFB_1_ and OTA in poultry feed (0.005 mg/kg for young poultry, 0.002 mg/kg for older, and 0.1 mg/kg, respectively) [32,33]. AFB_1_, OTA, and CIT are regulated in different foodstuffs but not in meat products, highlighting a regulatory gap [21].

Environmental conditions during processing, storage, and retail can lead to the contamination of meat surfaces with molds. Sources of contamination often include soil, water, equipment, handling, and transportation. A study by Darwish et al. [25] showed that inadequate freezing of chicken cuts and giblets, particularly those with high initial microbial loads, leads to mold growth. Frozen gizzards showed the highest mold count, followed by liver and breast. The prevalent genera included *Aspergillus*, *Penicillium*, *Cladosporium*, and *Alternaria*. The *Aspergillus* sp. identified—*Aspergillus niger*, *A. flavus*, *A. parasiticus*, and *Aspergillus versicolor*—are known to produce OTA, AF_S_, CPA, and STC [1,25].

In this study, mycotoxins were detected in 34% of all the analyzed edible chicken tissue samples. CPA was found in 20% of samples, with concentrations ranging from 2.56 to 6.50 µg/kg. OTA was present in 10% of samples, with levels between 0.18 and 0.51 µg/kg. AFB_1_ was also identified in 10% of samples, with concentrations ranging from 0.08 to 0.15 µg/kg. CIT was detected in 4% of samples, at levels ranging from 0.73 to 1.00 µg/kg (Figure S1). Other aflatoxins (B_2_, G_1_, and G_2_) and STC were not detected in any of the tested samples. Notably, CIT, OTA, and AFB_1_ were found exclusively in liver samples, suggesting that the liver may be a primary site for the accumulation of these mycotoxins. Liver samples showed a higher contamination frequency compared to muscle tissue, where only CPA was detected. On the other hand, a statistically significant higher concentration of CPA was observed in muscle tissue compared to liver tissue (*p* < 0.001). The detected mycotoxins were generally present at concentrations near the limits of detection and quantification. The results of the mycotoxin concentrations and their occurrence in chicken liver and breast muscle samples are shown in Table 2 and Table 3, respectively.

To the best of our knowledge, limited reports have documented the presence of mycotoxin contamination in chicken meat or meat products in general. When such data do exist, most of them pertain to aflatoxins or OTA to a lesser extent in chicken liver, primarily from non-European countries [23,26,28,34,35,36].

Darwish et al. [25] reported the concentration of AFs in frozen liver from Egypt, with levels reaching approximately 3.25 μg/kg. Similar concentrations were determined in a study by Iqbal et al. [23], where 35% of chicken samples from Pakistan tested positive for AF contamination, analyzed using HPLC. On the contrary, a study conducted by Wang et al. [28], which analyzed 70 chicken tissue samples from East China using LC-MS/MS, found no detectable AFB_1_ in chicken offal. Alaboudi et al. [26] detected AFB_1_ concentrations of 0.27 ± 0.06 μg/kg in liver/kidney samples from Jordan using LC-MS/MS. Additionally, Sineque et al. [37], employing the ELISA method, confirmed the liver as a primary target organ for AFs, detecting AFB_1_ in 39% of liver samples from Mozambique, with concentrations of 1.73 μg/kg. Amirkhizi et al. [27] also reported AFB_1_ contamination in 72% of liver samples from Iran, with concentrations ranging from 0.30 to 16.36 μg/kg. The concentrations of AFB_1_ in liver samples in the above-mentioned studies were found to be mostly 3 to 30 times higher than those reported in this study.

Darwish et al. [25] reported lower concentrations of AFB_1_ in frozen chicken breast compared to liver, with levels of 0.25 μg/kg. Similarly, Alaboudi et al. [26] detected AFB_1_ concentrations of 0.17 ± 0.03 μg/kg in chicken muscle samples from Jordan. In a study by Iqbal et al. [23], the highest AFB_1_ level in chicken breast was 1.19 μg/kg, while AFs reached 2.92 μg/kg; however, no AFs were detected in domestic chicken. These findings are consistent with the results of the present study in which no AFs were detected in chicken breast samples. This discrepancy underscores a significant difference in contamination levels between tissues, suggesting that the liver, as a primary target organ for AFs, tends to accumulate higher concentrations compared to other tissues.

The presence of AFB_1_ residues in poultry tissues such as the liver, as well as in other animal-derived products, has been directly linked to the contamination levels in poultry feed. Higher concentrations of AFB_1_ in feed result in increased tissue accumulation [37]. Conversely, Hussain et al. [38] reported that prolonged exposure to AFB_1_ enhances its elimination from the body, and that tissue contamination tends to decrease with the age of the birds.

Since AFB_1_ is found in edible chicken tissue, contamination with STC, as a precursor to AFB_1_, is also possible and should be reconsidered. The EFSA CONTAM Panel concluded that the available occurrence data were insufficient for a reliable human and animal dietary exposure assessment and emphasized the need for more occurrence data on STC in food and feed [6]. Although STC was not detected in the present study, it was found in our previous study of pork meat products, where concentrations ranged from 0.10 to 3.93 μg/kg in 4% of 250 analyzed samples. To the best of our knowledge, no other studies have reported on STC in meat and meat products. STC has also been reported in animal feed in previous studies [6,39]. Additionally, climate change in European countries is expected to increase the risk of STC contamination, as rising temperatures will create favorable conditions for the growth of *Aspergillu*s section *Versicolores* (optimal temperature 30 °C) and STC production (23–29 °C), potentially leading to higher concentrations of STC in the future [1,39].

The research results indicated, for the first time, the co-occurrence of the investigated mycotoxins in chicken liver samples. Although only 4 out of 17 contaminated samples contained more than one mycotoxin, two samples were found to contain both AFB_1_ and OTA, while the other two contained AFB_1_ and CPA. In the case of the latter combination, the potential co-occurrence has been recognized as plausible and warrants further investigation [7]. A study by Astoreca et al. [22] examined the production of CPA and aflatoxins by *A. flavus* strains isolated from maize, highlighting the potential for co-contamination in poultry feed. CPA has also been discussed in the context of mycotoxin effects on poultry and their productivity [40]. An analysis of meat from chickens orally dosed with 10 mg CPA/kg b.w. revealed that 14.5% of the administered dose remained in muscle tissue 48 h post-administration [41]. Currently, there are no available data on the occurrence of CPA in fresh meat, and the conducted study provides an initial insight into the presence of this mycotoxin in chicken meat, serving as a basis for future research. Although data on CPA occurrence are limited, our previous research conducted over the past 5 years has reported its presence in dry-cured meat products mostly in concentrations ranging from approximately 2.5 to 45 µg/kg, with a few extreme cases reaching up to 335 µg/kg [8,29,42]. In another study of meat products from Spain, levels ranged from 36 to 540 µg/kg [9]. In the present study, CPA was found with equal frequency in both liver and muscle tissues; however, its concentration was nearly twice as high in muscle. The prevalence of CPA in the meat products analyzed in this and our previous studies was similar, ranging between 13% and 20%.

OTA is recognized as a common contaminant in poultry feed [35] and the presence of OTA in meat products has been widely reported, particularly in dry-cured meat products where mold growth commonly occurs on the surface during production [8,30]. Rosa et al. [31] isolated *Aspergillus* and *Penicillium* sp. in high numbers from poultry feed samples, identifying *A. flavus* and *P. citrinum* as the most prevalent, with a high percentage of OTA producers detected, indicating a significant risk of mycotoxin transfer into the food chain.

Iqbal et al. [23] reported that 41% of chicken meat samples collected in Pakistan were contaminated with OTA, with the highest concentrations observed in liver samples (0.71–2.41 µg/kg). Al Khalaileh [35] found OTA contamination in 100% of liver and gizzard samples, with average concentrations of 5.86 µg/kg in the liver and 2.07 µg/kg in gizzard, as determined by ELISA. The highest levels were also detected in liver samples. These findings, consistent with the present study, highlight the liver as a primary site of OTA accumulation. Milicevic et al. [36] detected OTA in 20.7% of liver, kidney, and gizzard samples from chickens in Serbia, with concentrations ranging from 0.14 to 3.9 µg/kg in the liver and up to 9.94 µg/kg in gizzard and kidney tissues. The OTA concentrations in liver samples reported in these studies were approximately 2 to 15 times higher than those observed in the present work.

Al Khalaileh [35] detected OTA in 100% of thigh and leg samples and in 66.6% of breast meat samples from Jordan, with average concentrations of 2.61 µg/kg and 3.06 µg/kg, respectively. Iqbal et al. [23] reported OTA concentrations in chicken breast meat ranging from 0.28 to 0.81 µg/kg. In contrast, OTA was not detected in domestic chicken meat samples (wings, legs, and breast) in their study, which aligns with the findings of the present work for breast muscle. On the other hand, Murad [43] reported a higher prevalence and concentration of OTA in chicken breast and thigh meat samples compared to liver samples in products collected from supermarkets in Sulaimani, Iraq. OTA was found in 87% of the meat samples, with a mean concentration of 1.98 µg/kg, as determined by HPLC. Interestingly, these results contrast with other studies by showing higher OTA levels in muscle tissue than in liver. Guerrini et al. [44] detected OTA in chicken bile samples at concentrations ranging from 3.83 to 170.42 µg/L, concluding that contamination by OTA can occur in poultry.

Variations in mycotoxin contamination levels across studies reflect regional differences, which may be influenced by various factors, such as climatic conditions, feed management practices, and storage conditions in the countries of origin, as well as the analytical methods employed.

The possible co-occurrence of CIT and OTA has been reported in the literature, as both mycotoxins are produced by certain *Penicillium* sp., with *Penicillium verrucosum* being particularly significant [1,24]. *P. verrucosum* and *P. citrinum* have been identified with high prevalence in poultry feed samples in a study by Al Khalaileh [35]. In this study, only one sample was found to contain both OTA and CIT. According to the findings of Meerpoel et al. [45], although direct evidence of CIT accumulation in poultry meat remains limited, the possibility of residue presence cannot be excluded. Their study on the carry-over of CIT from feed to edible tissues in broiler chickens and laying hens revealed CIT concentrations ranging from 0.1 µg/kg in muscle to 70.2 µg/kg in liver. These findings indicate that CIT tends to accumulate in poultry tissues, potentially contributing to the total dietary CIT intake in humans. Some data from European countries highlight non-negligible exposure to CIT, particularly among children [24,46]. However, the specific dietary sources remain uncertain, due to the limited availability of comprehensive food analysis data for CIT. In a survey of food products from Belgian markets, fresh chicken meat, following cereal-based products, was among the food groups with the highest prevalence of CIT detection (74%), although concentrations were low, with a maximum of 0.23 µg/kg [24]. In contrast, in the present study, the concentration of CIT detected in the liver was approximately three times higher, although the occurrence was less frequent.

## 4. Conclusions

This study confirms the presence of the most important *Penicillium* and *Aspergillus* mycotoxins in edible chicken tissues such as breast muscle and liver, with contamination detected in 34% of the analyzed samples. The detected mycotoxins were generally present at concentrations near the limits of detection and quantification of the implemented analytical method and can be considered too low to pose an immediate health risk. However, their presence highlights the potential for increased contamination under future climate change conditions. The observed contamination rate suggests that further research into the occurrence of these mycotoxins in chicken and other poultry species may be necessary.

## Figures and Tables

**Table 1:** Method performance characteristics for mycotoxin analysis.

Mycotoxins	Linearity Range (μg/L)	LOD (μg/kg)	LOQ (μg/kg)	Recovery (%)	Matrix Effect (%)
OTA	0.2–5.0	0.18	0.59	119.4	1.8
AFB_1_	0.05–1.0	0.03	0.11	91.4	6.4
AFB_2_	0.05–1.0	0.03	0.11	95.1	6.5
AFG_1_	0.05–1.0	0.04	0.12	91.3	2.0
AFG_2_	0.05–1.0	0.05	0.17	89.1	11.6
CPA	0.5–25.0	2.45	8.07	97.5	1.9
CIT	1.0–10.0	0.60	1.98	100.9	0.7
STC	0.1–10.0	0.02	0.06	114.4	7.7

OTA—ochratoxin A; AFB_1_—aflatoxin B_1_; AFB_2_—aflatoxin B_2_; AFG_1_—aflatoxin G_1_; AFG_2_—aflatoxin G_2_; CPA—cyclopiazonic acid; CIT—citrinin; STC—sterigmatocystin; LOD—limit of detection; LOQ—limit of quantification.

**Table 2:** Concentration and prevalence of mycotoxins in chicken liver samples.

Mycotoxin	Concentration (µg/kg)			Occurrence	
	Lowest	Highest	Mean ± SD	No.	%
Aflatoxin B_1_	0.08	0.15	0.10 ± 0.04	5	20
Aflatoxin B_2_	<0.03	<0.03	NA	0	0
Aflatoxin G_1_	<0.04	<0.04	NA	0	0
Aflatoxin G_2_	<0.05	<0.05	NA	0	0
Sterigmatocystin	<0.02	<0.02	NA	0	0
Citrinin	0.73	1.00	0.86 ± 0.11	2	8
Ochratoxin A	0.18	0.51	0.35 ± 0.18	5	20
Cyclopiazonic acid	2.56	3.94	3.22 ± 0.57	5	20

Mean values are calculated from positive samples only (those > limit of detection); NA—not applicable; SD—standard deviation.

**Table 3:** Concentration and prevalence of mycotoxins in chicken breast muscle samples.

Mycotoxin	Concentration (µg/kg)			Occurrence	
	Lowest	Highest	Mean ± SD	No.	%
Aflatoxins (B_1_, B_2_, G_1_, G_2_)	<LOD	<LOD	NA	0	0
Sterigmatocystin	<0.02	<0.02	NA	0	0
Citrinin	<0.60	<0.60	NA	0	0
Ochratoxin A	<0.18	<0.18	NA	0	0
Cyclopiazonic acid	5.25	6.50	5.63 ± 0.59	5	20

Mean values are calculated from positive samples only (those > limit of detection); NA—not applicable; LOD—limit of detection; SD—standard deviation.

## Data Availability

The original contributions presented in this study are included in the article/Supplementary Materials. Further inquiries can be directed to the corresponding author.

## Supplementary Materials

The following supporting information can be downloaded at https://www.mdpi.com/article/10.3390/foods14122017/s1. Table S1: Sample information; Table S2: Instrumental settings for LC-MS/MS analysis; Figure S1: Chromatograms showing the natural occurrence of CPA, AFB1, OTA, and CIT at the highest detected concentrations in chicken breast muscle samples.
