# Supplementary material for: Mycotoxin Residues in Chicken Breast Muscle and Liver
Source: Foods. 2025 Jun 7;14(12):2017. doi: 10.3390/foods14122017 (PMC12192523; doi:10.3390/foods14122017)
Supplement: Supplementary file 1 [file foods-14-02017-s001.zip › foods-3669205 Suplementary data_Table S2 and Figure S1.pdf]

Table S2. Instrumental settings for LC-MS/MS analysis

Analyte	Precursor ion	Fragmentor voltage (V)	Product ions	Collision energy (eV)
CIT	251.1	110	233.1	15
			205.0	25
STC	325.1	130	310.0	25
			281.0	40
OTA	404.0	130	357.9	25
			239.0	10
CPA	337.2	110	196.3	25
			182.1	20
AFB1	313.1	170	285.1	23
			269.1	30
AFB2	315.1	170	287.1	25
			271.1	35
AFG1	329.1	170	283.0	25
			243.0	25
AFG2	331.1	170	256.9	32
			245.0	32

CIT—citrinin; STC—sterigmatocystin; OTA—ochratoxin A; CPA—cyclopiazonic acid; AFB_1—aflatoxin B_1; AFB_2—aflatoxin B_2; AFG_1—aflatoxin G_1; AFG_2—aflatoxin G_2;

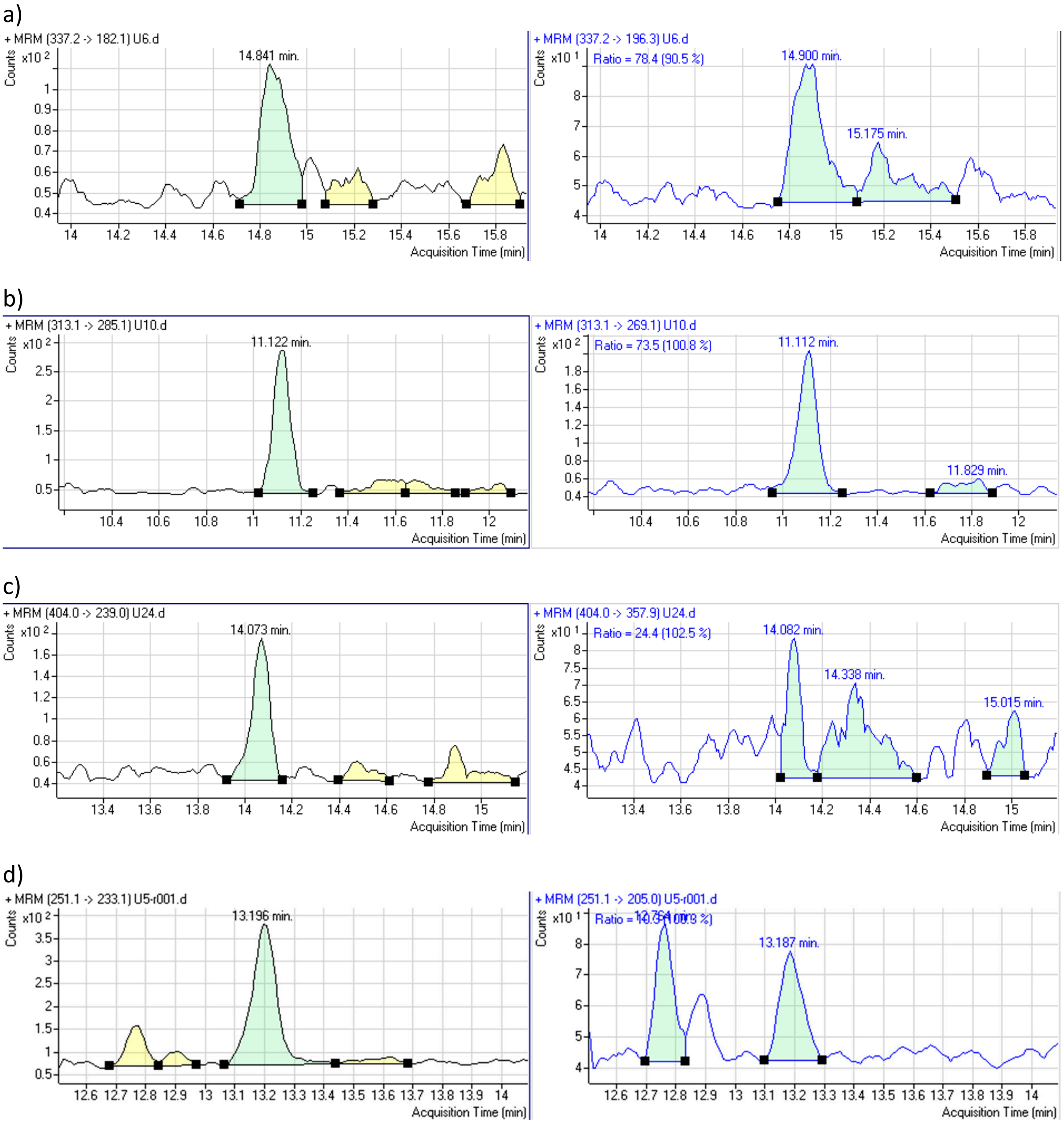

Figure S1. Chromatograms showing the natural occurrence of CPA, AFB_1, OTA, and CIT at the highest detected concentrations in chicken liver samples: a) CPA at 3.94 μg/kg; b) AFB_1 at 0.15 μg/kg; c) OTA at 0.51 μg/kg; d) CIT at 1.00 μg/kg
